# Photochemical Synthesis of *cis*,*trans*,*cis*‐1,2,3,4‐Tetrakis(diphenylphosphanyl)buta‐1,3‐diene and Its Metal Coordination

**DOI:** 10.1002/ejic.201800804

**Published:** 2018-11-08

**Authors:** Johannes Prock, Katharina Ehrmann, Wolfgang Viertl, Richard Pehn, Johann Pann, Helena Roithmeyer, Marvin Bendig, Alba Rodríguez Villalón, Holger Kopacka, Alexander Dumfort, Werner Oberhauser, Simon T. Clausing, Günther Knör, Peter Brüggeller

**Affiliations:** ^1^ Institute of General, Inorganic and Theoretical Chemistry Centrum for Chemistry and Biomedicine University of Innsbruck Innrain 80‐82, A ‐6020 Innsbruck Austria; ^2^ Istituto di Chimica dei Composti Organometallici (ICCOM‐CNR) Area di Ricerca CNR di Firenze via Madonna del Piano 10 50019 Sesto Fiorentino Italy; ^3^ Johannes Kepler University Linz Institute of Inorganic Chemistry Altenbergerstraße 69, A ‐4040 Linz Austria

**Keywords:** Phosphane ligands, Butadiene, Platinum, Photochemistry

## Abstract

The new bis(bidentate) tetraphosphane *cis*,*trans*,*cis*‐1,2,3,4‐tetrakis(diphenylphosphanyl)buta‐1,3‐diene (dppbd) (**7**) was obtained by applying a photochemical synthetic protocol. The key step of the photochemical reaction consisted of an intramolecular [2+2] cycloaddition involving a C–C double and triple bond of the Pt‐dimer species of the formula [Pt_2_Cl_4_(dppa)(*trans*‐dppen)] (**2**) {dppa = 1,2‐bis(diphenylphosphanyl)acetylene and dppen = 1,2‐bis(diphenylphosphanyl)ethene} leading to [Pt_2_Cl_4_(dppbd)] (**5**). The asymmetrically bridged precursor complex **2** was obtained by combinatorial chemistry. Single crystal X‐ray structure analyses of **2** and **5** proved that the intramolecular photochemical reaction occurred. Cyanolysis of **5** gave **7**, which was oxidized to dppbdO4 (**8**). Compounds **7**, **8**, and the Pd^II^ dimer complex [Pd_2_Cl_4_(dppbd)] (**9**) were characterized in the solid state by a single‐crystal X‐ray structure analysis. Interesting photophysial properties emerged from the UV/Vis spectra acquired for **7** and the dimer Os complexes *meso*‐Δ,Λ/Λ,Δ‐[Os_2_(bpy)_4_(dppbd)](PF_6_)_4_ (**10**) and *rac*‐Δ,Δ/Λ,Λ‐[Os_2_(bpy)_4_(dppbd)](PF_6_)_4_ (**11**).

## Introduction

The rational ligand design as a means of controlling properties of resulting complexes has proven to be mandatory to lift coordination chemistry to a new level. This control of ligand properties is largely accounted for by tuning steric and electronic characteristics of the ligand and is especially important for photoactive components of photosynthetic devices.^1^ In particular, multidentate ligands have become the focus of ongoing research.^2^ Homometallic polynuclear arrays increase the probability of light absorption and may prolong excited state lifetimes.[1a] Furthermore, multidentate ligands may be used to produce supramolecular photocatalysts combining the two tasks light‐harvesting and catalyzing proton reduction within one molecule.[2a] Multidentate ligands may also help make the chromophore more rigid and the central metal more sheltered from solvent molecules and therefore prevent quenching.^3^ Thereby push‐pull chromophores represent an especially interesting class of compounds in artificial photosynthesis for which direct electron transfer via a π‐spacer between donor and acceptor moiety is possible. The conjugated nature of the ligand is also conducive to the demands that the absorbance of the molecule be appropriately matched to the solar spectrum.^4^ Incorporation of main group elements, especially phosphorus, into the π‐skeleton provides a convenient method to fine‐tune the molecule's properties further.[1c]

Herein we describe the selective photochemical synthesis of *cis*,*trans*,*cis*‐1,2,3,4‐tetrakis(diphenylphosphanyl)buta‐1,3‐diene (**7**) as a novel tetraphos‐bridging‐ligand tailored for use in artificial photosynthesis. Thereby its stereoisomeric conformation decisively influences the ability to act as a bis‐bidentate ligand. Only a *cis*,*trans*,*cis* configuration ensures selective complex formation towards one main product. Furthermore, its conjugated nature gives rise to the hope that resulting complexes would utilize hopping electron transfer mechanisms and electron parking during artificial photosynthesis. The use of such ligands as bridges serving as switches has been discussed in literature. The conjugation may get lost due to conformational changes after electron transfer reactions. This would impair the reverse electron transfer reaction and deliver increased quantum yields.^5^, [1c], [1d]

To the best of our knowledge, only an *all trans* version of 1,2,3,4‐tetrakis(diphenylphosphanyl)buta‐1,3‐diene as a side product of the hydrophosphorylation of bis(diphenylphosphanyl)ethin with Ph_2_PH was obtained. The reaction mechanism remains unresolved in this case.^6^ We suspect that classical synthesis strategies are not suitable to obtain the *cis*,*trans*,*cis* configured ligand. Therefore, a photochemical approach was chosen to synthesize *cis*,*trans*,*cis*‐1,2,3,4‐tetrakis(diphenylphosphanyl)buta‐1,3‐diene.

## Results and Discussion

### Synthesis Protocol

**Scheme 1:**
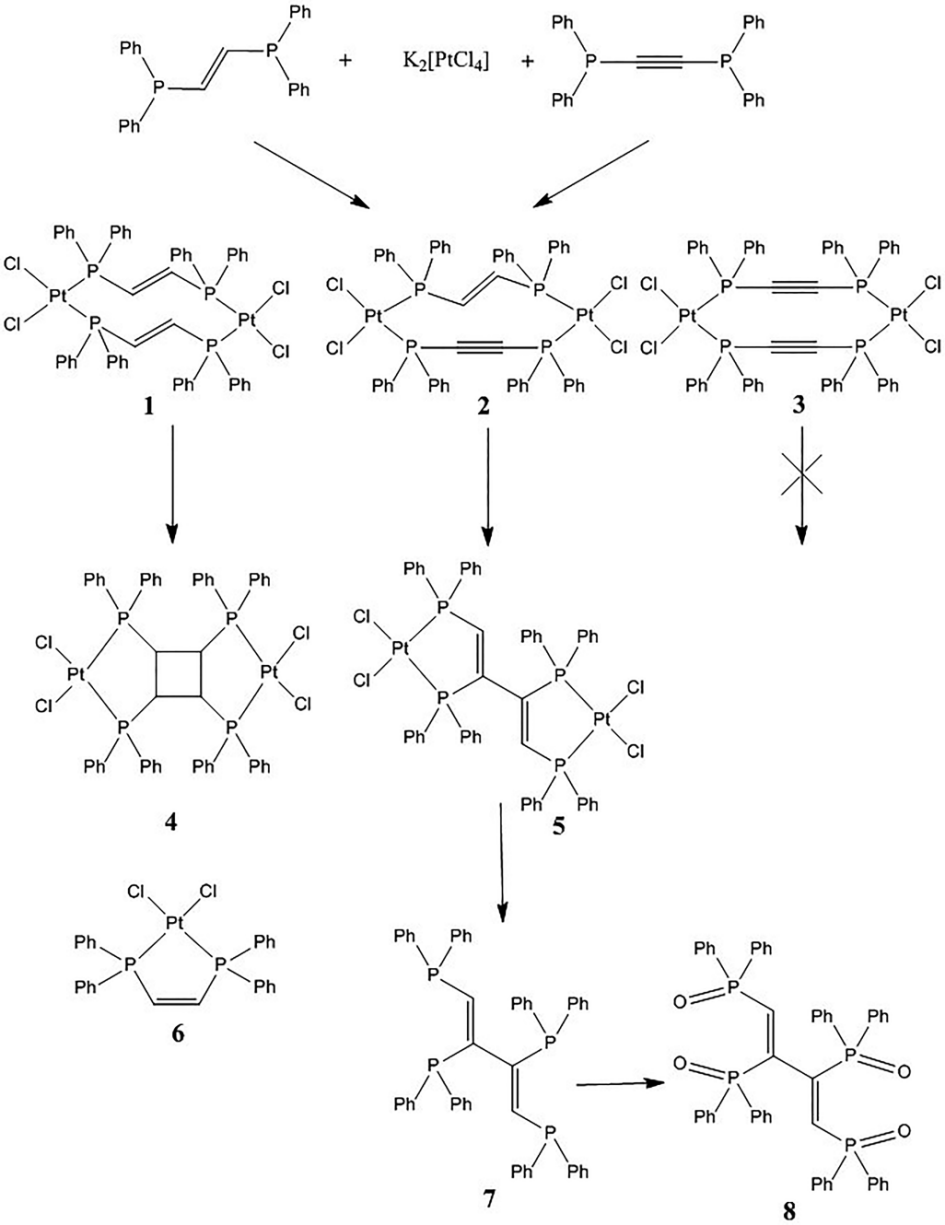
Syntheses of **1**–**8**.

**Scheme 2:**
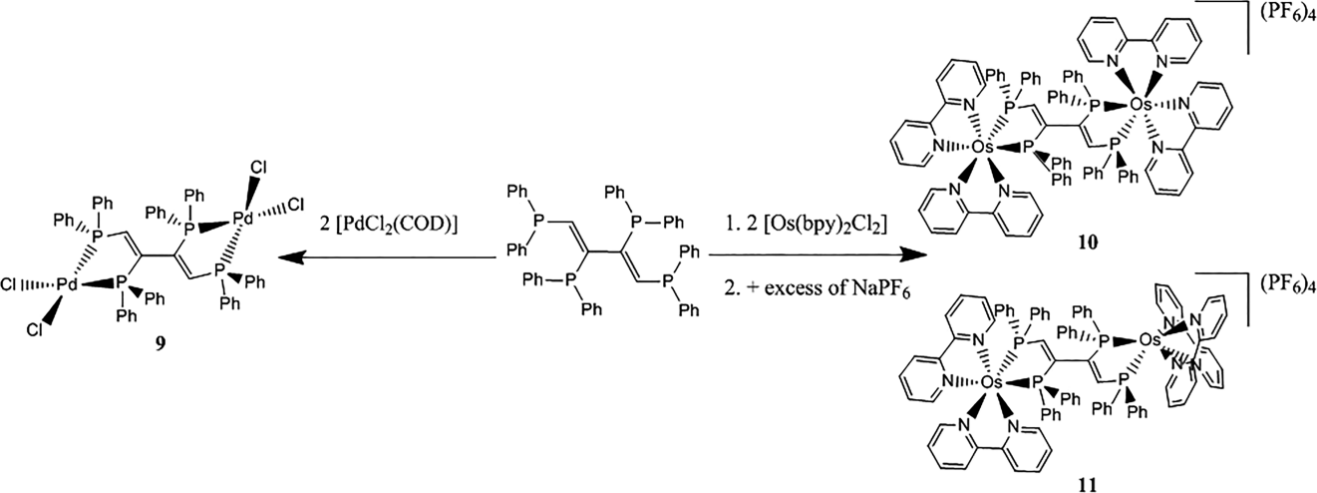
Syntheses of **9**–**11**.

The key species [Pt_2_Cl_4_(dppa)(*trans*‐dppen)] (**2**) (Scheme 1) where dppa is 1,2‐bis(diphenylphosphanyl)acetylene and *trans*‐dppen is *trans*‐1,2‐bis(diphenylphosphanyl)ethene, needed for the photochemical reaction was not obtained by a direct synthesis. Therefore another synthesis approach using combinatorial chemistry was pursued and a mixture of the compounds [Pt_2_Cl_4_(*trans*‐dppen)_2_] (**1**),^7^ **2** and [Pt_2_Cl_4_(dppa)_2_] (**3**)^8^ obtained. Due to the very fast formation and immediate precipitation of the products, this approach did not lead to an excess of the thermodynamically favored and undesired compounds **1** and **3** (Scheme 1). At this point it was not necessary to separate **1**–**3**. The photochemical reaction of **1** to give [Pt_2_Cl_4_(dppcb)] (**4**)^7^ occurs significantly faster than that of **2** to give [Pt_2_Cl_4_(dppbd)] (**5**) (Scheme 1). Exploiting the insolubility of **4** in CH_2_Cl_2_, it can be separated easily. **3** does not undergo any photocycloaddition reaction and can be separated from the reaction mixture, once **2** is completely converted to **5**. A typical side product of the photochemical synthesis of **4** is [PtCl_2_(*cis*‐dppen)] (**6**) where *cis*‐dppen is *cis*‐1,2‐bis(diphenylphosphanyl)ethene.^9^ **6** can also be easily separated from **5**. After the cyanolysis of **5** pure **7** was obtained in good yield. Simple oxidation of **7** by H_2_O_2_ led to *cis*,*trans*,*cis*‐1,2,3,4‐tetrakis(diphenylphosphanoyl)buta‐1,3‐diene (dppbdO_4_, **8**). The reaction of **7** with [PdCl_2_(COD)] gave [Pd_2_Cl_4_(dppbd)] (**9**, Scheme 2) in good yield, maintaining the *cis*,*trans*,*cis* configuration of **7**, as observed in **5**. Since dppbd has been tailored in order to generate interesting photophysical properties, due to its conjugated carbon‐backbone, the reaction of **7** with [Os(bpy)_2_Cl_2_] was also investigated. The two diastereoisomers *meso*‐Δ,Λ/Λ,Δ‐[Os_2_(bpy)_4_(dppbd)](PF_6_)_4_ (**10**) and *rac*‐Δ,Δ/Λ,Λ‐[Os_2_(bpy)_4_(dppbd)](PF_6_)_4_ (**11**) were obtained in excellent yield (Scheme 2) and separated from each other due to the insolubility of **10** in CH_2_Cl_2_.

**Scheme 3:**
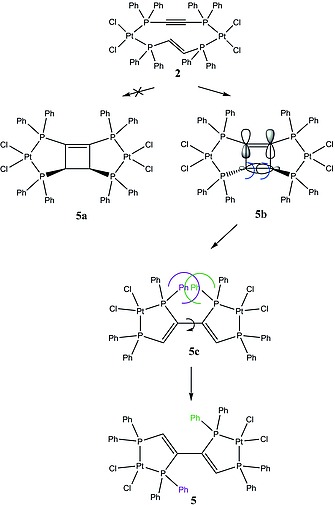
Reaction mechanism operative for the photochemical reaction of **2** to **5**.

Based on the obtained [2+2] photocycloaddition reaction product **5** (Scheme 1, its single‐crystal X‐ray structure is given below) and prior published work,^7^, ^10^ we propose the following reaction mechanism for this synthesis, as shown in Scheme 3. **2** undergoes a [2+2]‐photocycloaddition. Due to the presence of diphenylphosphanyl groups and the rather rigid molecular structure, **2** may foster not only a concerted but also a radical reaction mechanism. However, despite the inability to isolate the resulting cyclobutene species (**5b**) due to its instability, this intermediate is thought to exist as *trans* isomer only. This assumption can be justified through prior work where we found that the position of phosphorus substituents in reactants was conserved in the product of the photochemical cycloaddition.^7^, ^10^ While a radical cycloaddition mechanism involving bond cleavage would lead to a mixture of intermediates **5a** and **5b**, the concerted cycloaddition mechanism only allows the formation of intermediate **5b**.

The following ring opening reaction is likely to occur through a thermally induced conrotatory mechanism, which is in accordance with the Woodward–Hoffmann rule for simple cyclobutenes.^11^ Intermediate **5c** with *all cis*‐configuration is formed as a result of the ring opening which immediately rotates to give **5** avoiding hence steric clashes of the inner phosphorus substituents (see Scheme 3). The choice of the appropriate solvent for the photochemical reaction of **2** turned out to be critical. Coordinating solvents, such as DMF, led to ligand scrambling in **2** followed by the formation of the thermodynamically favored complexes **1** and **3** (Scheme 1). This ligand scrambling could be prevented by using toluene as solvent. Irradiating the solution of **1**–**3**, with a LED operating at 360 nm gave **5** in excellent yield. Furthermore, too high concentrations of reactants led to many side products. To the best of our knowledge similar reactions have been reported to occur only in the solid state.^12^ Notably, the replacement of Pt^II^ by Pd^II^ or Ni^II^ in the cycloaddition reaction gave not the desired product.

### Solid State Structures

**Figure 1:**
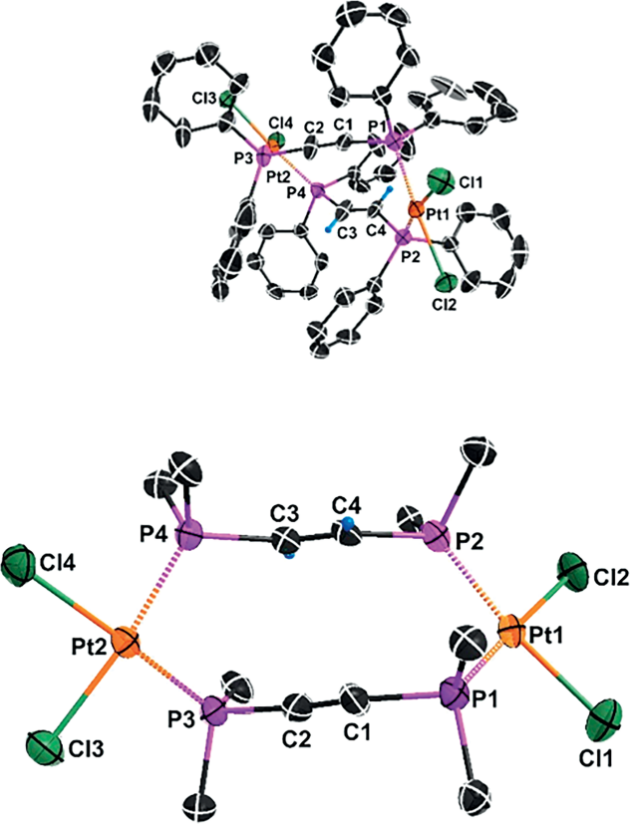
Simplified ORTEP diagrams of **2·**DMF. The hydrogen atoms of the phenyl groups and the DMF molecule are omitted for clarity (above). Only the *ipso* carbon atoms of the phenyl units are shown (below). The C1**···**C4 and C2**···**C3 contact distances are 3.050(9) Å and 3.104(9) Å, respectively.

**Figure 2:**
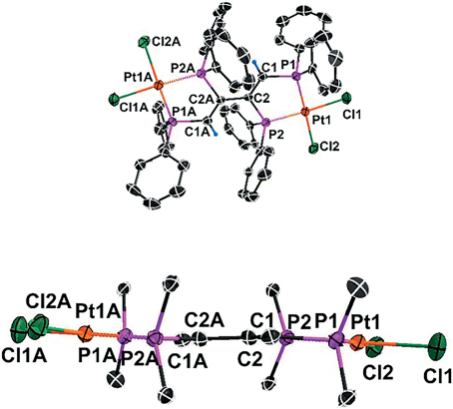
Simplified ORTEP diagrams of **5·**2DMF. The hydrogen atoms of the phenyl groups and DMF molecules are omitted for clarity (above). Only the *ipso* carbon atoms of the phenyl units are shown and all hydrogen atoms are omitted in order to demonstrate the nearly coplanar conformations of dppbd and the two square‐planar coordination units (below). A: –*x*, –*y*, –*z*.

The photochemical [2+2] cycloaddition of **2** to give **5** (Scheme 3) is proved by the single‐crystal X‐ray structure analyses of **2** and **5**. Different views of the two molecules are given in Figure 1 and Figure 2. Selected bond lengths and angles are presented in Table 1. The crystal structures of **2** and [Pt_2_Cl_4_(*trans*‐dppen)_2_] (**1**) are isomorphous,^7^ whereas [Pt_2_Cl_4_(dppa)_2_] (**3**) crystallizes in a different crystal system.^8^ The *trans*‐dppen and dppa bridges in **2** are twisted (Figure 1, above). Nevertheless, the C1**···**C4 and C2**···**C3 contact distances of 3.050(9) Å and 3.104(9) Å, respectively, favor the observed [2+2] photocycloaddition, making Pt^II^ a suitable template (Figure 1, below). At this point it is important to emphasize that neither Pd^II^ nor the less expensive Ni^II^ are useful templates for the synthesis of **7**.

**Table 1:** Selected bond lengths [Å] and angles [°] for compounds **2·**DMF and **5·**2DMF

Compound	**2·**DMF		**5·**2DMF	
Bond lengths [Å]	Pt1**···**Pt2	7.044(1)	Pt1**···**Pt1A^a^	8.016(1)
	Pt1–P1	2.218(2)	Pt1–P1	2.1938(16)
	Pt1–P2	2.254(2)	Pt1–P2	2.2118(16)
	Pt2–P3	2.228(2)		
	Pt2–P4	2.245(2)		
	Pt1–Cl1	2.331(2)	Pt1–Cl1	2.3436(17)
	Pt1–Cl2	2.349(2)	Pt1–Cl2	2.3584(17)
	Pt2–Cl3	2.322(2)		
	Pt2–Cl4	2.339(2)		
	C1–C2	1.171(10)	C1–C2	1.335(8)
	C3–C4	1.189(9)	C2–C2A	1.470(11)
				
Bond angles [°]	P1–Pt1–P2	97.43(7)	P1–Pt1–P2	87.73(5)
	P3–Pt2–P4	97.20(7)		
	Cl1–Pt1–Cl2	89.57(8)	Cl1–Pt1–Cl2	92.22(6)
	Cl3–Pt2–Cl4	87.18(8)		

a A: –*x*, –*y*, –*z*.

Interestingly, the C3–C4 double bond of 1.189(9) Å in **2** is shortened compared to *trans*‐dppen (Table 1). This stereoelectronic effect has been observed earlier in [PtCl_2_(*cis*‐dppen)] (**6**, Scheme 1)^9^ and could be a consequence of a favorable back‐bonding of two Pt^II^ centers in **2**.

The single‐crystal X‐ray structure of **5·**2DMF clearly reveals the desired *cis*,*trans*,*cis*‐configuration of dppbd (Figure 2, above). Also the bis(bidentate) coordination of **7** to Pt^II^ is obvious. A projection of **5** perpendicular to the butadiene backbone shows the nearly coplanar conformation of the dppbd bridge and the square‐planar coordination planes (Figure 2, below). As a consequence a strong delocalization of π electron density occurs throughout **5** and the central C2–C2A bond length is reduced to 1.470(11) Å (Table 1). A comparison of the structure parameters of **2** and **5** shows that a certain flexibility of both molecules seems to be necessary for a successful [2+2] photocycloaddition. Thus the Pt1**···**Pt2 distance of 7.044(1) Å in **2** is elongated to Pt1**···**Pt1A of 8.016(1) Å in **5**. Both Pt–P bond lengths of **5** are shortened compared to the corresponding parameters in **2**, which is certainly a consequence of the five‐membered ring effect only present in **5** (Table 1). Due to the formation of five‐membered rings during the [2+2] photocycloaddition, the P1–Pt1–P2 and P3–Pt2–P4 bite angles of 97.43(7)° and 97.20(7)°, respectively, in **2·**DMF are reduced to 87.73(5)° in **5·**2DMF (Table 1).

**Figure 3:**
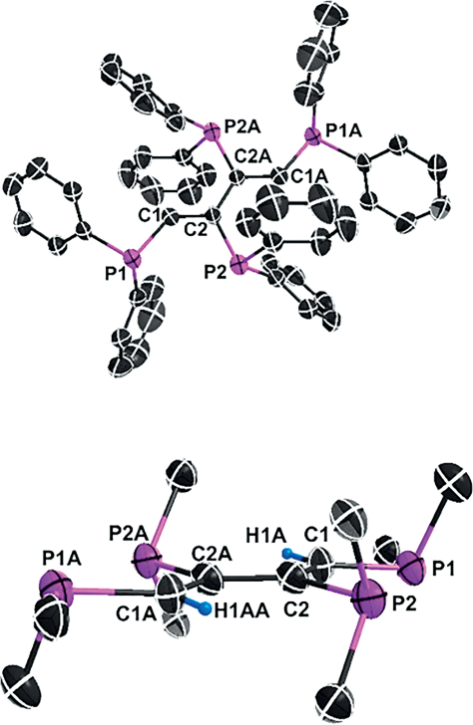
Simplified ORTEP diagrams of **7**. All hydrogen atoms are omitted for clarity (above). Only the *ipso* carbon atoms of the phenyl units are shown in order to demonstrate the planar conformation of dppbd (below). A: –*x*, –*y*, –*z*. In the asymmetric unit of the crystal lattice of **7** a second conformation of dppbd is present. It is nearly identical to the one shown in this Figure.

**Figure 4:**
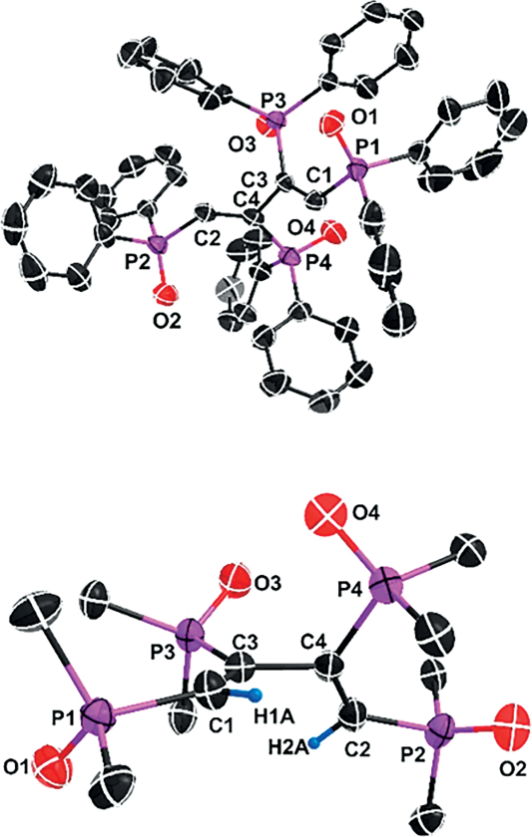
Simplified ORTEP diagrams of **8**. All hydrogen atoms are omitted for clarity (above). Only the *ipso* carbon atoms of the phenyl units are shown in order to demonstrate the non‐planar conformation of dppbdO_4_ (below).

In order to study the solid state properties of **7** and its complete oxidation product dppbdO_4_ (**8**), the corresponding single‐crystal X‐ray structures have been determined. Different views of the two molecules are given in Figure 3 and Figure 4. Selected bond lengths and angles are presented in Table 2. The asymmetric unit of **7** consists of two conformations of dppbd, which are nearly identical. Therefore, only one conformer is shown in Figure 3. The phosphorus atoms and the butadiene bridge are coplanar (Figure 3, below). As a result the C1–C2–C2A–C1A torsion angle calculated as Newman projection along C2–C2A is 180° also due to crystallographic constraints. The same is true for the corresponding parameter of the second conformer of **7**. In fact, as in **5** strong delocalization of π electron density throughout **7** brings about a reduction of the central C2–C2A and C4–C4A bond lengths in both conformers to 1.491(4) Å and 1.489(4) Å, respectively (Table 2) and an absorption in the visible range of the light spectrum (i.e. yellow color of **7**, vide infra). By contrast, the conformation of dppbdO_4_ (**8**) is completely different (see Figure 4). Obviously the four oxygen atoms are orientated in a way to prevent unfavorable repulsive interactions (Figure 4, above). As a consequence the four phosphorus atoms and the butadiene backbone are no more coplanar as in the case of **7** (Figure 4, below). Therefore, the C1–C3–C4–C2 torsion angle calculated as Newman projection along C3–C4 is only 116.0° and the reduction of the central C3–C4 bond length to 1.508(3) Å (Table 2) is less pronounced than in **5** and **7**. Obviously, the strong delocalization of π electron density observed in **5** and **7** is now interrupted in **8**. However, a conjugated backbone is necessary in case of energy and/or electron transfer via superexchange to occur. This means that dppbd is a ligand that is capable of acting as bridge as a switch for future applications.[5a]

**Table 2:** Selected bond lengths [Å] and angles [°] for compounds **7**–**9**

Compound	**7**		**8**	
Bond lengths [Å]	P1–C1	1.821(2)	P1–C1	1.806(2)
	P2–C2	1.843(2)	P2–C2	1.800(2)
	P3–C3	1.814(2)	P3–C3	1.843(2)
	P4–C4	1.848(2)	P4–C4	1.841(2)
	C1–C2	1.344(3)	C1–C3	1.333(3)
	C2–C2A	1.491(4)	C2–C4	1.339(3)
	C3–C4	1.342(3)	C3–C4	1.508(3)
	C4–C4A	1.489(4)		
				
Bond angles [°]	C1–C2–C2A	120.9(3)	C1–C3–C4	118.71(18)
	P1–C1–C2	128.73(19)	C3–C4–C2	117.44(18)
	P2–C2–C1	115.15(17)	P1–C1–C3	132.63(17)
	C3–C4–C4A	121.8(2)	P3–C3–C1	130.98(16)
	P3–C3–C4	127.72(18)	P2–C2–C4	132.55(17)
	P4–C4–C3	114.04(17)	P4–C4–C2	131.47(16)
Compound	**9**			
Bond lengths [Å]	Pd1**···**Pd1A^a^	8.054(1)		
	Pd1–P1	2.2069(11)		
	Pd1–P2	2.228(1)		
	Pd1–Cl1	2.3475(11)		
	Pd1–Cl2	2.3580(12)		
	C1–C2	1.339(4)		
	C2–C2A	1.487(6)		
				
Bond angles [°]	P1–Pd1–P2	87.05(3)		
	Cl1–Pd1–Cl2	95.50(4)		

a A: –*x*, –*y*, –*z*.

The crystal structures of **5·**2DMF and [Pd_2_Cl_4_(dppbd)]**·**2DMF (**9·**2DMF, Scheme 2) are isomorphous. These two structures fulfil the criteria of the same ligands, the same coordination number and geometry, isomorphous crystal lattices and equal experimental conditions and therefore allow comparison of the radii of Pd and Pt atoms.^13^ The mean M–P bond length of 2.2028(11) Å in **5·**2DMF is significantly smaller for Pt than for Pd of 2.2175(7) Å in **9·**2DMF. Assuming a covalent radius of tetra‐coordinate phosphorus as r(P)_cov_ = 1.11 Å,^14^ the covalent radii of square planar d^8^ Ni, Pd, and Pt are estimated as 1.06, 1.17 and 1.16 Å, respectively. The results presented here are also in full agreement with the smaller covalent radius for Au than for Ag, a phenomenon which is generally referred to as the “relativistic or Lanthanoide contraction”.^15^ As a consequence the Pt1**···**Pt1A distance of 8.016(1) Å in **5·**2DMF is significantly smaller than the corresponding Pd1**···**Pd1A parameter of 8.054(1) Å in **9·**2DMF.

### Solution Structures

In order to confirm the solid‐state structures of **2**, **5** and **7**–**11** in solution multinuclear NMR spectroscopy, mass spectrometry, UV/Vis spectroscopy and excitation/emission spectroscopy including lifetime determination of the excited state have been applied. The ^195^Pt {^1^H} NMR spectrum of **2** shows a doublet of doublets at –4444.5, clearly indicating two inequivalent phosphorus atoms, while in the corresponding ^31^P{^1^H} NMR spectrum the signal at *δ* = 5.78 ppm is attributed to the *trans*‐dppen bridge by comparison with the symmetrically bridged compound [Pt_2_Cl_4_(*trans*‐dppen)_2_] (**1**).^7^ Furthermore, this latter signal shows a ^4^ *J*_PtP_ coupling of 50.0 Hz also typical for **1**. The second ^31^P peak of **2** at –12.98 ppm is attributed to dppa.^8^ Both ^31^P signals show ^1^ *J*_PtP_ coupling constants of 3414.0 and 3684.0 Hz, respectively, in line with a *trans* P–Pt–Cl arrangement.^7^ In the ^13^C{^1^H} spectrum the signals of the bridging carbon atoms are clearly separated from the aromatic ones. The ^1^H NMR spectrum reveals the two hydrogen atoms of *trans*‐dppen at *δ* = 5.86 ppm. They are split into a triplet due to ^2^ *J*_PH_ + ^3^ *J*_PH_ of 19.7 Hz. Together with the [M – Cl]^+^ peak in the positive FAB mass spectrum this clearly indicates that the solution structure of **2** is in accordance with its solid‐state structure. After the [2+2] photocycloaddition the solubility of **5** is reduced compared to **2** making ^195^Pt {^1^H} and ^13^C{^1^H} NMR spectra impossible. The ^31^P{^1^H} NMR spectrum of **5** shows two singlets at *δ* = 66.60 and 36.20 ppm, where the high‐field signal is attributed to the outer PPh_2_‐groups (see Figure 2). P_outer_ and P_inner_ in **5** show comparable coordination shifts (vide infra). The ^1^ *J*_PtP_ coupling constants of 3652.0 and 3562.0 Hz, are again in line with a *trans* P‐Pt‐Cl arrangement.^7^ In the ^1^H NMR spectrum two equivalent butadiene protons are shown at *δ* = 5.82 ppm, clearly indicating that the inversion center located in between the two halves of the molecule is retained in solution (Figure 2). The observation of the [M – Cl]^+^ peak in the positive FAB mass spectrum again confirms the result of the single‐crystal X‐ray structure of **5** and its solution structure.

**Figure 5:**
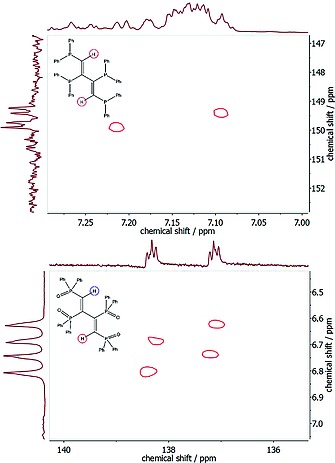
Top spectrum: **7** recorded with BIRD‐HMQC (^1^H NMR spectrum horizontal); bottom spectrum: **8** recorded with HETCOR (^1^H NMR spectrum vertical). The two different ^13^C{^1^H}, ^1^H correlation methods were used in order to obtain the best results.

The single‐crystal X‐ray structure of **7** obtained after cyanolysis of **5** shows two nearly identical conformations (vide supra). Due to the dynamic solution behavior of **7** at room temperature, no multinuclear NMR indication of these two conformations can be found in solution. Also upon cooling a CH_2_Cl_2_ solution of **7** down to –80 °C did not give any hint in the ^31^P{^1^H} NMR spectra of a second conformation of **7** in solution. A ^31^P{^1^H} NMR spectrum of **7**, acquired at room temperature, shows two doublets at –3.14 and –22.11 ppm. The high‐field doublet is attributed to the outer PPh_2_‐groups (Figure 3), and its chemical shift is comparable to that of *cis*‐dppen (i.e. –23.0 ppm). Due to the presence of lone electron pairs in **7** the ^3^ *J*(P,P) parameter of 138.5 Hz is rather high.^7^ In the ^13^C{^1^H} spectrum the signals of the butadiene carbon atoms are clearly separated from the aromatic ones. They occur at 149.60 and 139.21 ppm as doublet of doublets due to the ^1^ *J*_PC_ coupling constants of 37.1 and 11.4 Hz and ^2^ *J*_PC_ coupling constants of 12.6 and 5.0 Hz, respectively. However, the butadiene ^1^H NMR signals are obscured by the aromatic ones. In order to make an assignment possible a ^13^C{^1^H}, ^1^H correlation spectrum of **7** in CD_2_Cl_2_ was obtained (Figure 5, top). It clearly reveals a doublet for the two butadiene hydrogen atoms centered at *δ* = 7.16 ppm showing a ^2^ *J*_PH_ of 36.0 Hz. Furthermore, the ^13^C{^1^H} NMR signal at *δ* = 149.60 ppm was assigned to the bridging carbon atoms that are connected to a hydrogen atom. The multiplicities of the ^31^P{^1^H}, ^13^C{^1^H}, and ^1^H NMR signals are completely in line with a planar, centrosymmetric structure of **7** in solution corresponding to that of the solid state (Figure 3).

However, the ^31^P{^1^H} NMR spectrum of **8** consists of two doublets of doublets at *δ* = 28.26 and 16.55 ppm showing notable roof effects, corresponding to an AA′BB′ spin system. The AA′ part at *δ* = 28.26 ppm is again attributed to the inner phosphorus atoms of dppbdO_4_ (see Figure 4). It is split by ^3^ *J*_PP_ of 10.8 and 5.9 Hz, respectively. The outer phosphorus atoms of the BB′ part are split by the ^3^ *J*_PP_ coupling of 10.8 Hz and a ^4^ *J*_PP_ of 5.5 Hz. In the ^13^C{^1^H} NMR spectrum of **8** four poorly resolved doublets of doublets at 156.69, 155.54, 138.32, and 137.14 ppm are observed (i.e. carbon atoms of the butadiene carbon bridge), clearly indicating the chemical inequivalence of these carbon atoms. The ^1^H NMR spectrum of **8** shows for the bridge hydrogen atoms a doublet of doublets at *δ* = 6.73 ppm (i.e. ^2^ *J*_PH_ 35.1 Hz and ^3^ *J*_PH_ of 19.0 Hz). The ^13^C{^1^H}, ^1^H correlation spectrum of **8** in CD_2_Cl_2_ (Figure 5, bottom) reveals that the high field carbon atoms at 138.32 and 137.14 ppm are connected to these protons. In the case of **8** the multiplicities of the ^31^P{^1^H} and ^13^C{^1^H} NMR signals clearly indicate that a simple planar, centrosymmetric structure is not possible. Obviously, the main conformation in solution corresponds to the single crystal solid‐state structure, showing the inequivalence of the phosphorus and carbon atoms (Figure 4). However, in the ^1^H NMR spectrum the chemical shift differences of the two protons are too small to be resolved.

**Figure 6:**
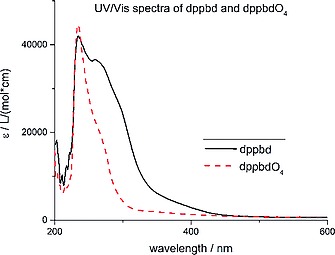
UV/Vis spectra of 1.0 × 10^–5^ m MeCN solutions of **7** (black line) and **8** (red dashed line) at 25 °C.

This is confirmed by the ^1^H NMR spectrum of [Pd_2_Cl_4_(dppbd)] (**9**), where both butadiene hydrogen atoms are assigned to a singlet at *δ* = 5.84 ppm (i.e. conformation with inversion center, as observed for the solid‐state structure). The ^31^P{^1^H} NMR spectrum of **9** shows two doublets at *δ* = 90.27 and 57.71 ppm split by ^2^ *J*_PP_ + ^3^ *J*_PP_ of 15.0 Hz, where the high‐field signal is attributed to the outer PPh_2_‐groups like in the case of **5**. P_outer_ and P_inner_ in **9** show again comparable coordination shifts. The crystal structures of **5·**2DMF and **9·**2DMF are isomorphous. Furthermore, also the UV/Vis spectra of **7** and **8** are in agreement with their conformational differences (Figure 6). In the latter compound the peak at 230 nm stems from π→π* transitions of the phenyl groups, since delocalization of electron density throughout the butadiene bridge is interrupted as a consequence of its nonplanar conformation. Therefore, **8** appears as a white powder. However, a slight shoulder at 275 nm already indicates that a second type of π→π* transitions occur which are attributed to the double bonds of butadiene.[2b] These transitions are strongly enhanced in **7** as a result of the strong delocalization of electron density throughout the butadiene bridge including all four coplanar phosphorus atoms (Figure 3). Since also the lone electron pairs at the phosphorus atoms are involved in this delocalization, a pronounced tailing of these aliphatic double bond π→π* transitions reaches beyond 400 nm towards even higher wavelengths (Figure 6). As a consequence, compound **7** is a yellow powder. This on/off delocalization clearly demonstrates that **7** is capable of serving as bridge as a switch depending on its conformation in related complexes.[5a]

**Figure 7:**
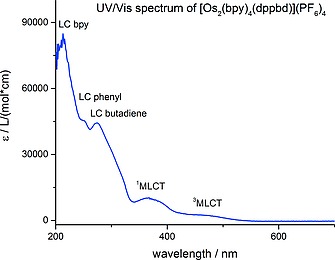
UV/Vis spectrum of a 1.0 × 10^–5^ m MeCN solution of a 1:1 mixture of **10** and **11** at 25 °C. The LC absorptions can be unequivocally assigned due to comparison with Figure 6.

**Figure 8:**
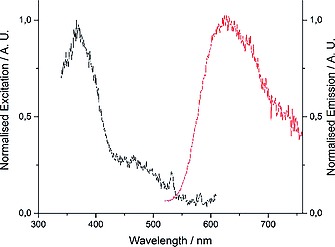
Excitation/emission spectra for a 1.0 × 10^–5^ m MeCN solution of a 1:1 mixture of **10** and **11** at 25 °C. The emission spectrum was recorded for an excitation at 375 nm, the excitation spectrum for an emission at 630 nm.

**Figure 9:**
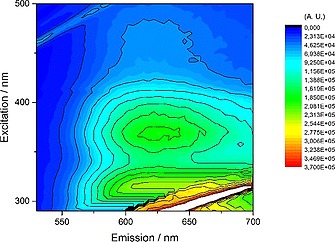
3D‐contour plot of the combined excitation/emission spectra for a 1.0 × 10^–5^ m MeCN solution of a 1:1 mixture of **10** and **11** at 25 °C. The ^3^MLCT emission centered at 630 nm is excitable from 300 to 500 nm. The feature in the bottom right corner belongs to Rayleigh scattering.

**Figure 10:**
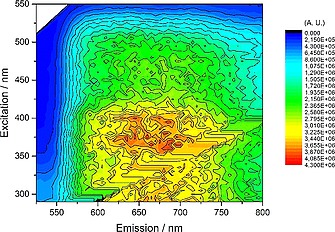
3D‐contour plot of the combined excitation/emission spectra for a 1.0 × 10^–5^ m EtOH/MeOH/MeCN (v/v = 4:1:2) cryogenic solution of a 1:1 mixture of **10** and **11** at –196 °C. The lack of non‐radiative decay at this temperature reveals the manifold of ^3^MLCT states leading to emissions from 550 to 800 nm.

The reaction between [Os(bpy)_2_Cl_2_] and dppbd conducted in a 2:1 molar ratio (Scheme 2) gave a 1:1 mixture of the dimer Os complexes, *meso*‐ΔΛ,ΛΔ‐[Os_2_(bpy)_4_(dppbd)](PF_6_)_4_ (**10**) and *rac*‐ΔΔ,ΛΛ‐[Os_2_(bpy)_4_(dppbd)](PF_6_)_4_ (**11**). The corresponding single‐crystal X‐ray structures have been reported recently.^16^ The UV/Vis spectrum of a 1:1 mixture of these two diastereoisomers is shown in Figure 7. As shown above for **7** and **8** the ligand centered (LC) π→π* transitions at 230 nm and 275 nm are assigned to the phenyl groups and butadiene, respectively. They are typical for a planar conformation of dppbd in agreement with both single‐crystal X‐ray structures of **10** and **11**.^16^ A further LC π→π* transition at 215 nm is attributed to the bipyridine groups. The transitions at 375 nm and 475 nm belong to ^1^MLCT and ^3^MLCT states, respectively.[2b] The excitation spectrum of these diastereoisomers perfectly matches the UV/Vis spectrum (compare Figure 7 and Figure 8) indicating the purity of the compounds. However, from both MLCT states only one single emission centered at 630 nm at 298 K is observable showing the typical tailing towards longer wavelengths (Figure 8). The 3D‐contour plot in Figure 9 clearly reveals that this emission at 630 nm is excitable from 300 to 500 nm. This could be due to ISC effects and has been observed earlier.^17^ At 77 K these effects are strongly enhanced and the manifold of ^3^MLCT states show emissions from 550 to 800 nm (Figure 10). At ambient temperature the luminescence lifetime of 199(4) ns for a 1:1 mixture of **10** and **11** is comparable to the corresponding parameter of 243(8) ns for the homodimetallic analog [Os_2_(dppcb)(bpy)_4_]^4+^.^17^

The use of the new tetraphosphane ligand dppbd in **10** and **11** has produced a chromophore with extraordinary stability.^16^ This is certainly a consequence of the bis(bidentate) coordination behavior of dppbd and the resulting five‐membered chelate rings. Furthermore, planar conformations of dppbd as shown in the single crystal structures of **10** and **11** lead to a further stabilization due to an excessive delocalization of electron density throughout the butadiene backbone and the adjacent phosphorus atoms. Thus, combined with a water‐soluble matrix‐stabilized palladium nanoparticle catalyst an excellent and unprecedented stability of 45 days was obtained in the photocatalytic hydrogen generation from water. Further work combining dppbd with different metals suitable for chromophores is in progress.

## Conclusions

The novel bis(bidentate) tetraphosphane *cis*,*trans*,*cis*‐1,2,3,4‐tetrakis(diphenylphosphanyl)buta‐1,3‐diene (dppbd) has been synthesized by using a combined photochemical and combinatorial synthetic protocol, which was revealed to be the only synthetic method to obtain it in the *cis*,*trans*,*cis*‐configuration. The conjugated carbon‐backbone of dppbd might allow superexchange of electrons possibly controlled by a bridge as a switch conformational effect in these future dyads. Favorable entropic effects provided by an excess of the free dppbd may pave the way to obtain heterodimetallic species and dyads upon reaction of [MCl_2_(dppbd)] (M = Ni^2+^, Pd^2+^ and Pt^2+^) with a second metal center.

## Experimental Section

**Reagents and General Procedures:** The ligands dppa [1,2‐bis(diphenylphosphanyl)acetylene] and *trans*‐dppen [*trans*‐1,2‐bis(diphenylphosphanyl)ethene], K_2_[PtCl_4_], [PdCl_2_(COD)], NaCN and H_2_O_2_ were purchased from Aldrich and [Os(bpy)_2_Cl_2_] from Colonial Metals. Dry solvents of purissimum grade were used for all syntheses, spectroscopic measurements, and crystallization purposes. A Schlenk apparatus and oxygen‐free, dry Ar were utilized in the syntheses of all complexes, when working with unprotected phosphanes. Solvents were degassed by several freeze‐pump‐thaw cycles prior to use. As irradiation source a UVAHAND LED 405 nm, 70 watts by Hoenle UV Technology was used.

**Instrumentation:** Fourier‐mode ^195^Pt{^1^H}, ^31^P{^1^H}, ^13^C{^1^H} and ^1^H NMR spectra were obtained using a Bruker DPX‐300 spectrometer (internal deuterium lock) at 298 K. Positive chemical shifts are downfield from the standards: 1.0 m Na_2_PtCl_6_ for ^195^Pt{^1^H}, 85 % H_3_PO_4_ for ^31^P{^1^H} and TMS for ^13^C{^1^H} and ^1^H chemical shifts, given in ppm. FAB‐MS spectra were obtained on a Finnigan MAT‐95 spectrometer, using 3‐nitrobenzylalcohol (NOBA) as matrix. Elemental analyses were carried out using a Perkin–Elmer Model 2400 C,H,N elemental analyzer.

**Syntheses of 1–3:** To a solution of K_2_[PtCl_4_] (2.00 g, 4.82 mmol) in water (40 mL) were added a solution of dppa (0.950 g, 2.41 mmol) and *trans*‐dppen (0.955 g, 2.41 mmol) in dichloromethane (30.0 mL). Subsequently, 90 mL of ethanol were added in order to achieve complete miscibility. The mixture was vigorously stirred for 90 min resulting in a colorless suspension. The suspension was then concentrated to a volume of 50.0 mL by means of a vacuum and the remaining slurry was filtered off. The solid white residue was washed with water and dried by vacuum. A mixture of **1**, **2** and **3** in a 1:2:1 molar ratio (i.e. based on ^31^P{^1^H} NMR integration) was obtained. Yield (**1–3**): 3.12 g (98 %). Pure compound **2** can be separated from the above mixture via partial crystrallization in DMF. Single crystals suitable for an X‐ray structure analysis with the composition **2·**DMF were obtained by slow evaporation of a solution of **2** in DMF at room temperature. C_55_H_49_Cl_4_NOP_4_Pt_2_ (1395.79): calcd. C 47.33, H 3.54; found C 47.23, H 3.59 %. Melting point > 350 °C. Mass spectrometry: FAB pos., NOBA, *m/z* = 1287.34 [M – Cl]^+^. NMR spectroscopic data for **2**: ^31^P{^1^H} NMR (121.49 MHz, CD_2_Cl_2_, 25 °C): *δ* = 5.78 (dd, ^1^ *J*_PtP_ = 3414.0, ^4^ *J*_PtP_ = 50.0, ^2^ *J*_PP_ = 9.0, ^3^ *J*_PP_ = 7.0 Hz, 2 P, P_*tran*s‐dppen_), –12.98 (dd, ^1^ *J*_PtP_ = 3684.0, ^2^ *J*_PP_ = 9.0, ^3^ *J*_PP_ = 7.0 Hz, 2 P, P_dppa_) ppm. ^1^H NMR (300.0 MHz, CD_2_Cl_2_, 25 °C): *δ* = 7.15–7.70 (m, 40 H, Ph‐*H*), 5.86 (t, ^2^ *J*_PH_ + ^3^ *J*_PH_ = 19.7 Hz, 2 H, C*H*_bridge_) ppm. ^13^C{^1^H} NMR (75.476 MHz, CD_2_Cl_2_, 25 °C): *δ* = 138.0–140.0 (m, 4 C, *C*_bridge_), 125.0–134.0 (m, 48 C, Ph‐*C*) ppm. ^195^Pt{^1^H} NMR (64.22 MHz, CD_2_Cl_2_, 25 °C): *δ* = –4444.5 (dxd) ppm.

**Synthesis of 5:** The dry mixture of **1**–**3** was split into three parts of 500.0 mg each and suspended in toluene (120.0 mL), followed by irradiation which lasted 4 h. The obtained solid was filtered off and suspended in 50.0 mL of dichloromethane. The suspension was ultrasonicated for 15 min, followed by stirring for 2 hours, filtering and washing with dichloromethane giving **4**. The remaining filtrate consisting of **2** and **3** was concentrated to dryness and the obtained solid again suspended in 120.0 mL of a (5:1, v:v) solvent mixture of toluene and dichloromethane, after having split the solid mixture in three parts. The suspensions were irradiated for seven days and the obtained solid filtered off. The filtrate mainly contained the side product **6**, which was removed. The combined solids were suspended in dichloromethane (40.0 mL), treated with ultrasound for 10 min and then stirred for one hour. Then toluene (20.0 mL) was added and the white solid filtered off and dried under vacuum. Yield: 800.0 mg (71 % based on the 1:2:1 mixture of **1**–**3**). Single crystals, suitable for an X‐ray structure analysis of **5·**2DMF were obtained by slow evaporation of a DMF solution of **5** at room temperature. C_58_H_56_Cl_4_N_2_O_2_P_4_Pt_2_ (1468.89): calcd. C 47.42, H 3.84; found C 47.33, H 3.89 %. Melting point > 350 °C. Mass spectrometry: FAB pos., NOBA, *m/z* = 1287.24 [M – Cl]^+^. NMR spectroscopic data for **5**: ^31^P{^1^H} NMR (121.497 MHz, CD_2_Cl_2_, 25 °C): *δ* = 66.60 (s, ^1^ *J*_PtP_ = 3652.0 Hz, 2P, P_inner_), 36.20 (s, ^1^ *J*_PtP_ = 3562.0 Hz, 2 P, P_outer_) ppm. ^1^H NMR (300.0 MHz, [D_7_]DMF, 25 °C): *δ* = 7.20–7.74 (m, 40 H, Ph‐*H*), 5.82 (s, 2 H, C*H*_bridge_) ppm.

**Synthesis of 7:** To a suspension of **5** (500.0 mg, 0.378 mmol) in dichloromethane (30.0 mL) was added a solution of NaCN (2.00 g, 40.8 mmol) in water (30.0 mL). Subsequently ethanol (90.0 mL) was added to the suspension, causing an immediate color change to yellow. The reaction mixture was stirred for 12 hours, followed by reduction of the original volume to 60.0 mL via evaporation. The obtained yellow solid was filtered off, washed with water (100.0 mL) and recrystallized from dichloromethane and petroleum ether. The yellow recrystallized powder was dried in vacccum. Single crystals suitable for an X‐ray structure analysis of **7** were obtained by slow evaporation of a dichloromethane solution of **7** under an atmosphere of argon at room temperature. Yield: 269.0 mg (90 %). C_52_H_42_P_4_ (790.74) calcd. C 78.98 H, 5.35; found C 78.89, H 5.39 %. Melting point: 161 °C. Mass spectrometry: FAB pos., NOBA, *m/z* = 791.85 [M + H]^+^. ^31^P{^1^H} NMR (121.497 MHz, CD_2_Cl_2_, 25 °C): *δ* = –3.14 (d, ^3^ *J*_PP_ = 138.5 Hz, 2P, P_inner_), –22.11 (d, ^3^ *J*_PP_ = 138.5 Hz, 2P, P_outer_) ppm. ^1^H NMR (300.0 MHz, CD_2_Cl_2_, 25 °C): *δ* = 7.16 (d, ^2^ *J*_PH_ = 36.0 Hz, 2 H, C*H*_bridge_), 6.30–7.26 (m, 40 H, Ph‐*H*) ppm. ^13^C{^1^H} NMR (75.476 MHz, CD_2_Cl_2_, 25 °C): *δ* = 149.60 (dd, ^1^ *J*_CP_ = 37.1, ^2^ *J*_CP_ = 12.6 Hz, 2C, *C*H_bridge_), 139.21 (dd, ^1^ *J*_CP_ = 11.4, ^2^ *J*_CP_ = 5.0 Hz, 2C, *C*_bridge_), 135.45 + 132.78–132.97 + 128.17–128.33 (Ph‐*C*) ppm.

**Synthesis of 8:** To a solution of **7** (50.0 mg, 0.063 mmol) in DMF (5.0 mL) was added a H_2_O_2_ solution (35.0 wt.‐% in H_2_O, 1.0 mL) and the resulting mixture was stirred for 30 min giving a colorless solution, which was successively concentrated to dryness, the residue suspended in water (10.0 mL) and the obtained white solid filtered off, washed three‐times with water (3.0 mL) and dried by vacuum. Suitable crystals for a single‐crystal X‐ray analysis of **8·**CH_2_Cl_2_ **·**0.25H_2_O were obtained by slow evaporation of a dichloromethane solution of **8** under air at room temperature. This crystallization was only successful under an atmosphere of air, since water molecules are incorporated into the crystal lattice. Yield: 48.0 mg (89 %). C_53_H_44.5_Cl_2_O_4.25_P_4_ (944.17) calcd. C 67.42 H, 4.75; found C 67.33, H 4.79 %. Melting point: 191.0 °C. Mass spectrometry: FAB pos., NOBA, *m/z* = 855.85 [M + H]^+^. ^31^P{^1^H} NMR (121.497 MHz, CD_2_Cl_2_, 25 °C): *δ* = 28.26 (dd, ^3^ *J*_PP_ = 10.8 and 5.9 Hz, 2 P, P_inner_), 16.55 (dd, ^3^ *J*_PP_ = 10.8, ^4^ *J*_PP_ = 5.5 Hz, 2 P, P_outer_) ppm. ^1^H NMR (300.0 MHz, CD_2_Cl_2_, 25 °C): *δ* = 7.95–8.02 (m, 8 H, Ph‐*H*), 7.22–7.42 (m, 32 H, Ph‐*H*), 6.73 (dd, ^2^ *J*_PH_ = 35.1, ^3^ *J*_PH_ = 19.0 Hz, 2 H, C*H*_bridge_) ppm. ^13^C{^1^H} NMR (75.476 MHz, CD_2_Cl_2_, 25 °C): *δ* = 156.69 (dd, 1 C, *C*_bridge_), 155.54 (dd, 1 C, *C*_bridge_), 138.32 (dd, 1 C, *C*H_bridge_), 137.14 (dd, 1 C, *C*H_bridge_), 128.4–134.8 (Ph‐*C*) ppm. The doublet of doublets of the butadiene ^13^C{^1^H} NMR resonances are only poorly resolved.

**Synthesis of 9:** To a solution of **7** (30.0 mg, 0.038 mmol) in 5.0 mL of dichloromethane was added a solution of [PdCl_2_(COD)] (21.6 mg, 0.076 mmol) in 3.0 mL of dichloromethane. After 2 h stirring, the solvent was removed by evaporation and the resulting white powder washed with diethyl ether and dried by vacuum. Single crystals, suitable for an X‐ray structure analysis of **9·**2DMF were obtained by slow evaporation of a DMF solution of **9** at room temperature. Yield: 30.4 mg (70 %). C_58_H_56_Cl_4_N_2_O_2_P_4_Pd_2_ (1291.53) calcd. C 53.94, H 4.37; found C 53.89, H 4.41 %. Melting point > 350 °C. Mass spectrometry: FAB pos., NOBA *m/z* = 1109.89 [M – Cl]^+^. ^31^P{^1^H} NMR (121.497 MHz, [D_7_]DMF, 25 °C): *δ* = 90.27 (d, ^2^ *J*_PP_ + ^3^ *J*_PP_ = 15.0 Hz, 2 P, P_inner_), 57.71 (d, ^2^ *J*_PP_ + ^3^ *J*_PP_ = 15.0 Hz, 2 P, P_outer_) ppm. ^1^H NMR (300.0 MHz, [D_7_]DMF, 25 °C): *δ* = 7.29–7.80 (m, 40 H, Ph‐*H*), 5.84 (s, 2 H, C*H*_bridge_) ppm.

**Synthesis of the 1:1 Mixture of 10 and 11:** To a solution of [Os(bpy)_2_Cl_2_] (81.6 mg, 0.142 mmol) in a (1:3, v:v) solvent mixture of ethoxyethanol and ethyleneglycol (6.0 mL) was added dppbd (56.2 mg, 0.071 mmol) at room temperature. The reaction mixture was refluxed at 140 °C for two hours by means of an oil bath. The reaction mixture was then cooled to room temperature and upon addition of NaPF_6_ (129.0 mg, 8.0 mmol) the product precipitated and was then filtered off and washed with a small portion of CH_2_Cl_2_. The obtained red powder was a (1:1) mixture of the diastereomers (**10** and **11**). Recrystallization of the obtained powder from CH_2_Cl_2_ was necessary to remove impurities (i.e. 2–4 % of mononuclear Os complex). Yield: 163.63 mg (97 %). C_92_H_74_F_24_N_8_P_8_Os_2_ (2375.855) calcd. C 46.51, H 3.14, N 4.72; found C 46.48, H 3.18, N 4.70 %. Melting point > 350 °C. Mass spectrometry: FAB pos., NOBA *m/z* = 2230.87 [M – PF_6_]^+^. ^31^P{^1^H} NMR (121.497 MHz, CD_2_Cl_2_): *δ* = 45.75 (d, ^2^ *J*_PP_ = 5.5 Hz, 2 P, P_*meso*,inner_), 44.29 (d, ^2^ *J*_PP_ = 5.5 Hz, 2 P, P_*rac*,inner_), 22.10 (d, ^2^ *J*_PP_ = 5.5 Hz, 2 P, P_*rac*,outer_), 20.75 (d, ^2^ *J*_PP_ = 5.5 Hz, 2 P, P_*meso*,outer_) ppm. ^1^H NMR (300.0 MHz, [D_3_]MeCN, 25 °C): *δ* = 8.52–7.73 (m, 32 H, Bpy‐*H*), 7.71 (d, ^2^ *J*_PH_ = 1.4 Hz, 2 H, C*H*_bridge_), 7.4–6.1 (m, 40 H, Ph‐*H*) ppm for the *meso*‐form and *δ* = 8.50–5.3 (m, 32 H, Bpy‐*H* + m, 40 H, Ph‐*H*), 7.53 (d, ^2^ *J*_PH_ = 1.3 Hz, 2 H, C*H*_bridge_) ppm for the *rac*‐form.

**Crystallography:** Single‐crystal X‐ray diffraction analyses of **2·**DMF, **5·**2DMF, **7**, **8·**CH_2_Cl_2_ **·**0.25H_2_O, and **9·**2DMF were performed on a Nonius Kappa CCD diffractometer (Mo‐*K_α_*‐tube) with the use of combined ϕ–ω‐scans. Denzo and Scalepack programs were utilized for data collection, cell refinement, data reduction and empirical absorption corrections. Structural refinement was performed with the SHELXL‐2014/7 program. Final refinements on *F* ^2^ were carried out with anisotropic thermal parameters for all non‐hydrogen atoms in all cases. The hydrogen atoms were included using a riding model with isotropic *U* values depending on the *U*_eq_ of the adjacent carbon atoms. In the case of **8** the hydrogen atoms on the solvent molecules have been omitted due to substantial disorder in these molecules.

CCDC 1849603 (for **2**), 1849604 (for **5**), 1849605 (for **7**), 1849606 (for **8**), and 1849607 (for **9**) contain the supplementary crystallographic data for this paper. These data can be obtained free of charge from The Cambridge Crystallographic Data Centre.

## Supporting information

Supporting Information
