# Supplementary material for: Photochemical Synthesis of cis,trans,cis‐1,2,3,4‐Tetrakis(diphenylphosphanyl)buta‐1,3‐diene and Its Metal Coordination
Source: Eur J Inorg Chem. 2018 Nov 8;2018(46):4962–71. doi: 10.1002/ejic.201800804 (PMC6471117; doi:10.1002/ejic.201800804)
Supplement: Supplementary file 1 — Supporting Information [file EJIC-2018-4962-s001.pdf]

European Journal of Inorganic Chemistry

Supporting Information

Photochemical Synthesis of *cis,trans,cis*-1,2,3,4-Tetrakis(diphenylphosphanyl)buta-1,3-diene and Its Metal Coordination

Johannes Prock, Katharina Ehrmann, Wolfgang Viertl, Richard Pehn, Johann Pann, Helena Roithmeyer, Marvin Bendig, Alba Rodríguez Villalón, Holger Kopacka, Alexander Dumfort, Werner Oberhauser,* Simon T. Clausing, Günther Knör,* and Peter Brüggeller*

ejic201800804-sup-0001-SupMat.pdf

Contents

Compounds 2

$^{31}P\{^{1}H\}$-NMR Spectroscopy 2

$^{195}Pt\{^{1}H\}$-NMR Spectroscopy 9

Compounds

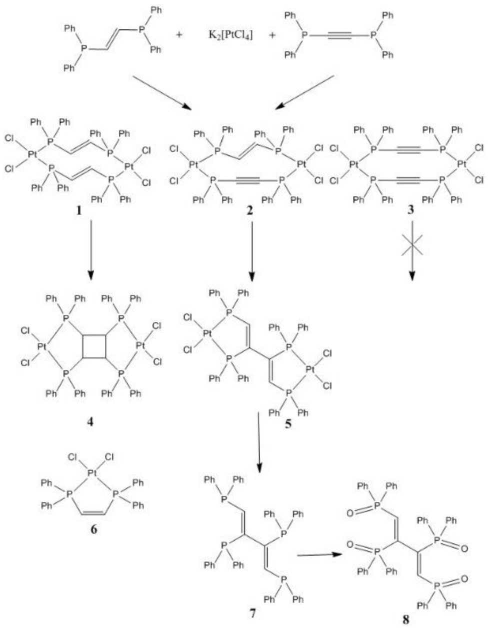

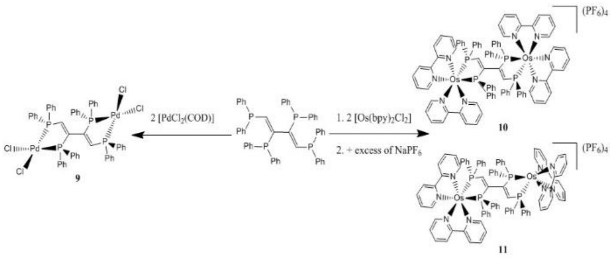

Figure S1: Structures of discussed compounds.

$^{31}P\{^1H\}$-NMR Spectroscopy

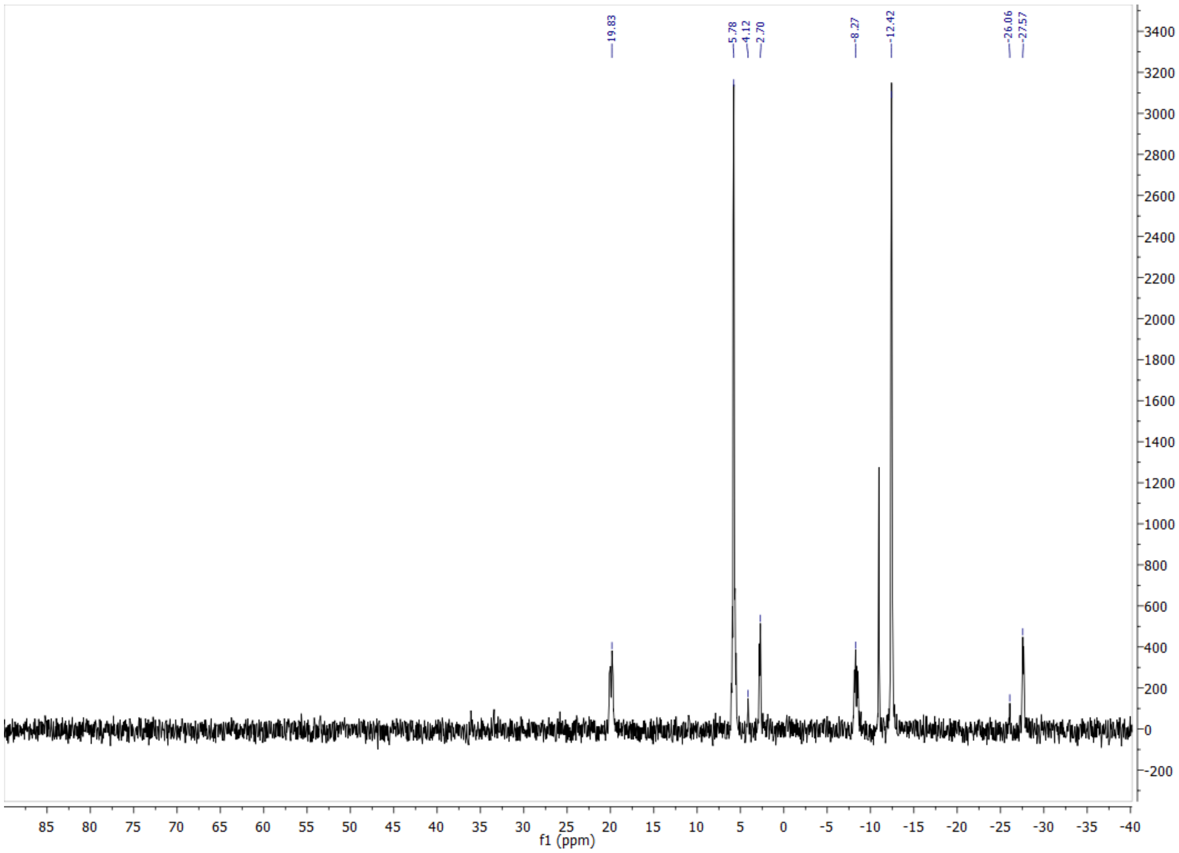

*Figure S1: $^{31}P\{^1H\}$-NMR spectrum of **2**, containing a small amount of **3** in CH_2Cl_2 at ambient temperature.*

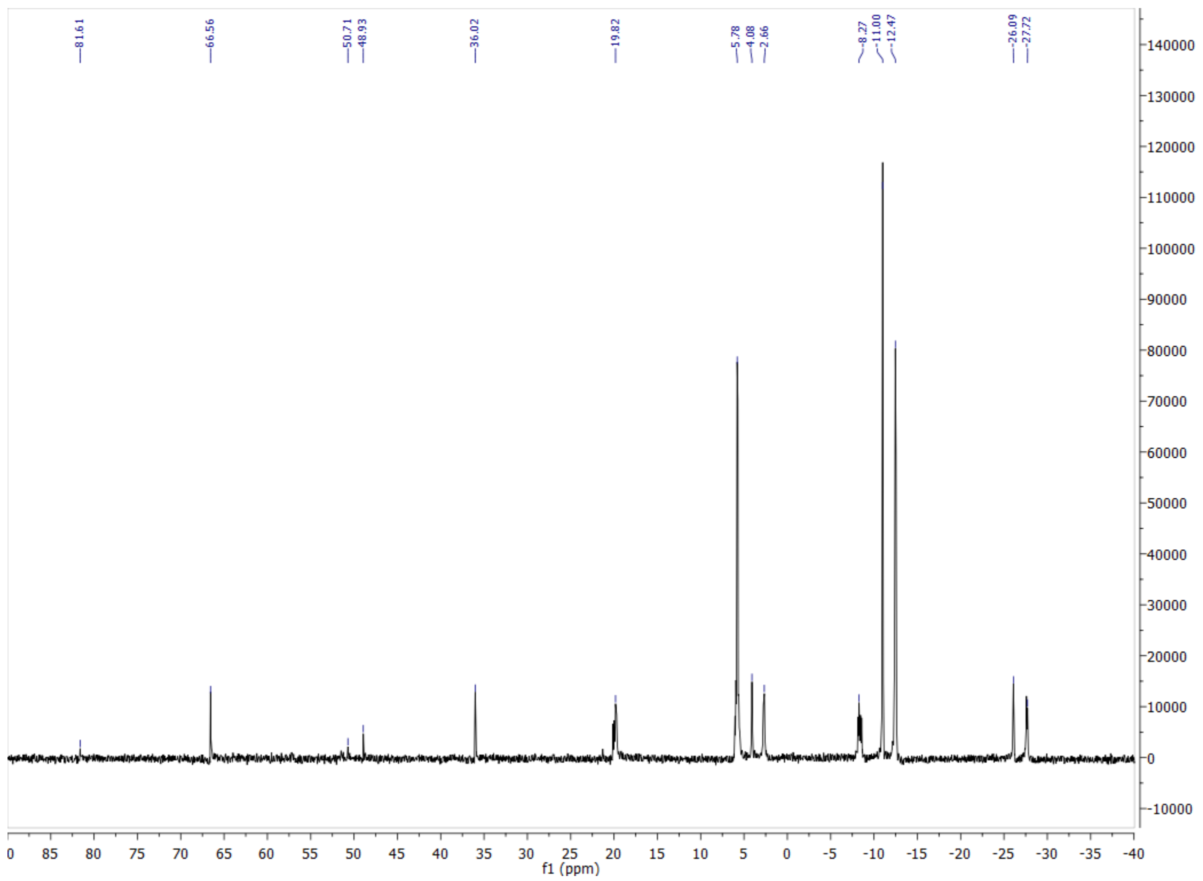

*Figure S2: $^{31}P\{^{1}H\}$-NMR of the mixture of **1, 2** and **3** during irradiation in CH_2Cl_2.*

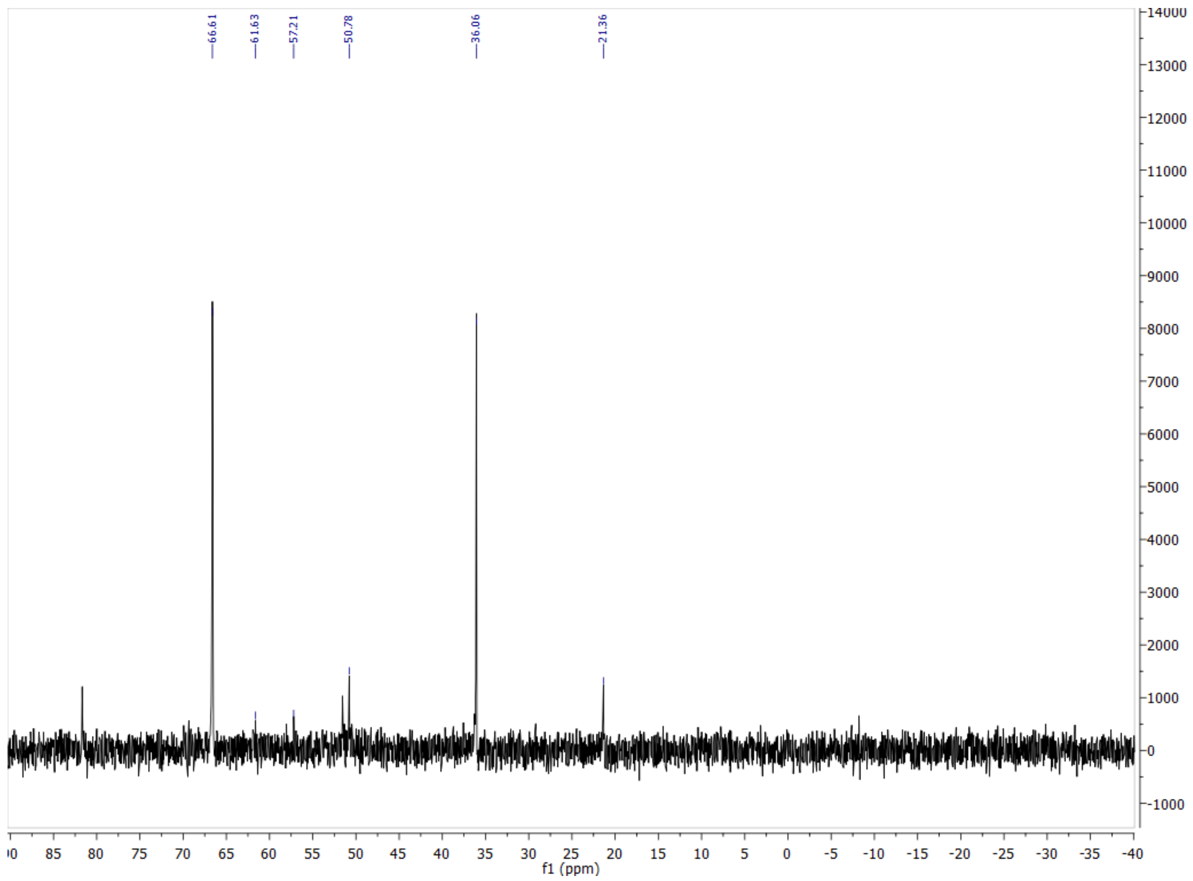

*Figure S3: $^{31}P\{^{1}H\}$-NMR of **5** in CH_2Cl_2.*

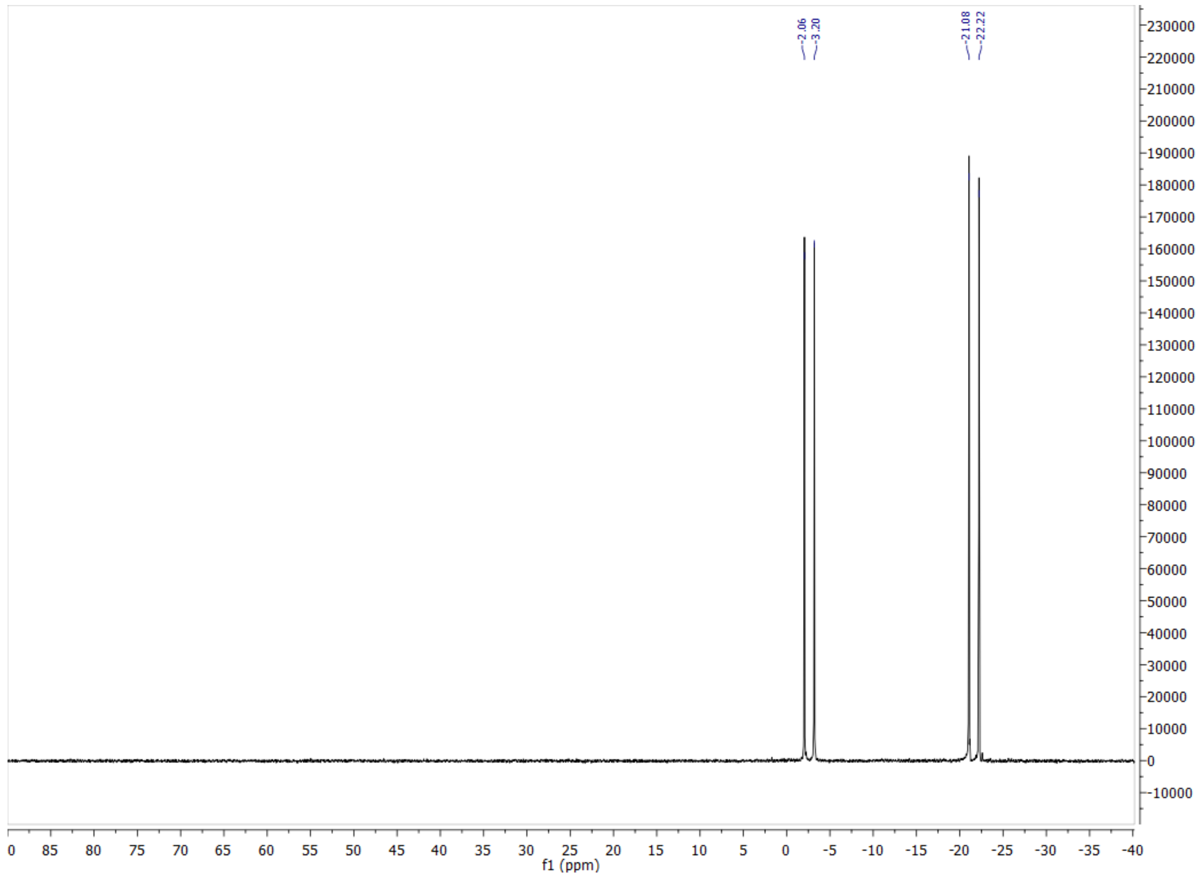

*Figure S4: $^{31}P\{^{1}H\}$-NMR of **7** in CH_2Cl_2.*

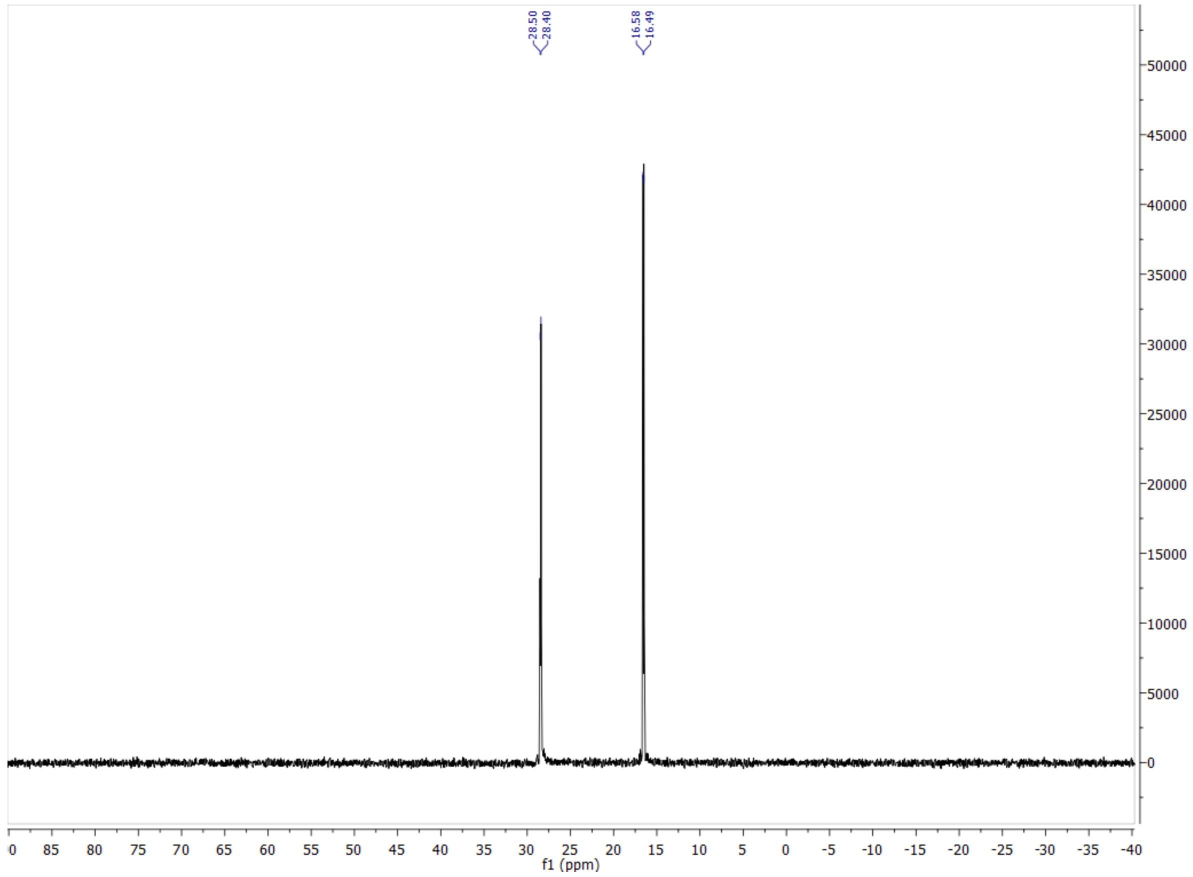

*Figure S5: $^{31}P\{^{1}H\}$-NMR of **8** in CH_2Cl_2.*

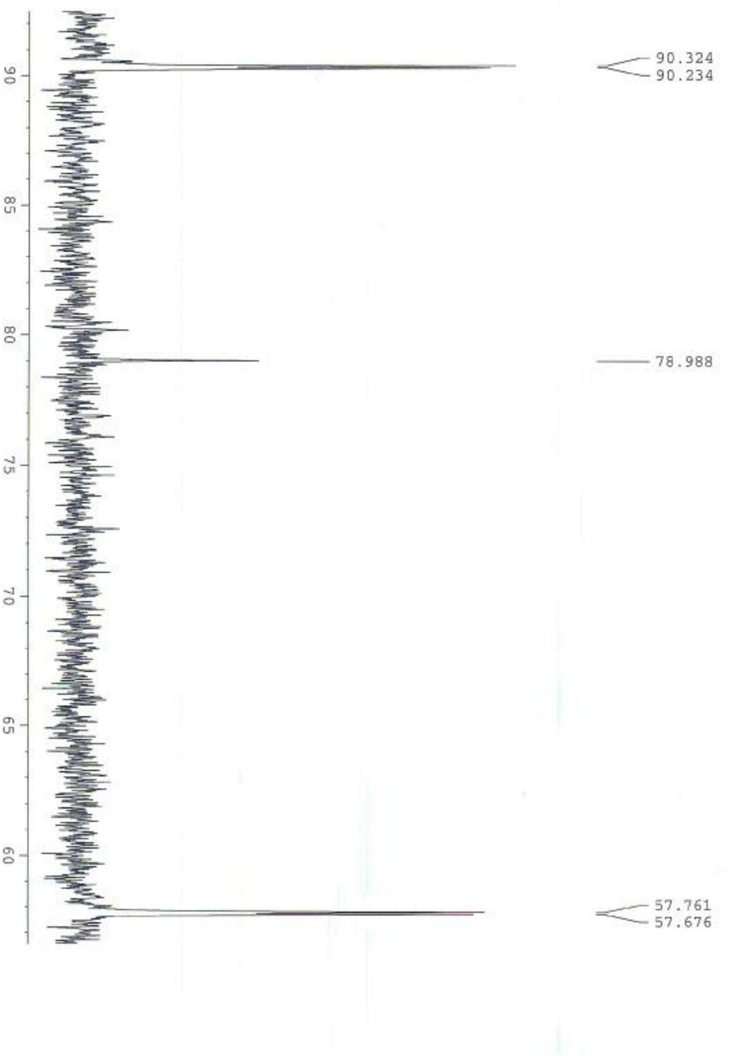

*Figure S6: $^{31}P\{^1H\}$-NMR spectrum of **9** in CH_2Cl_2. The peak at about 79 ppm is a small unknown impurity removable by recrystallisation.*

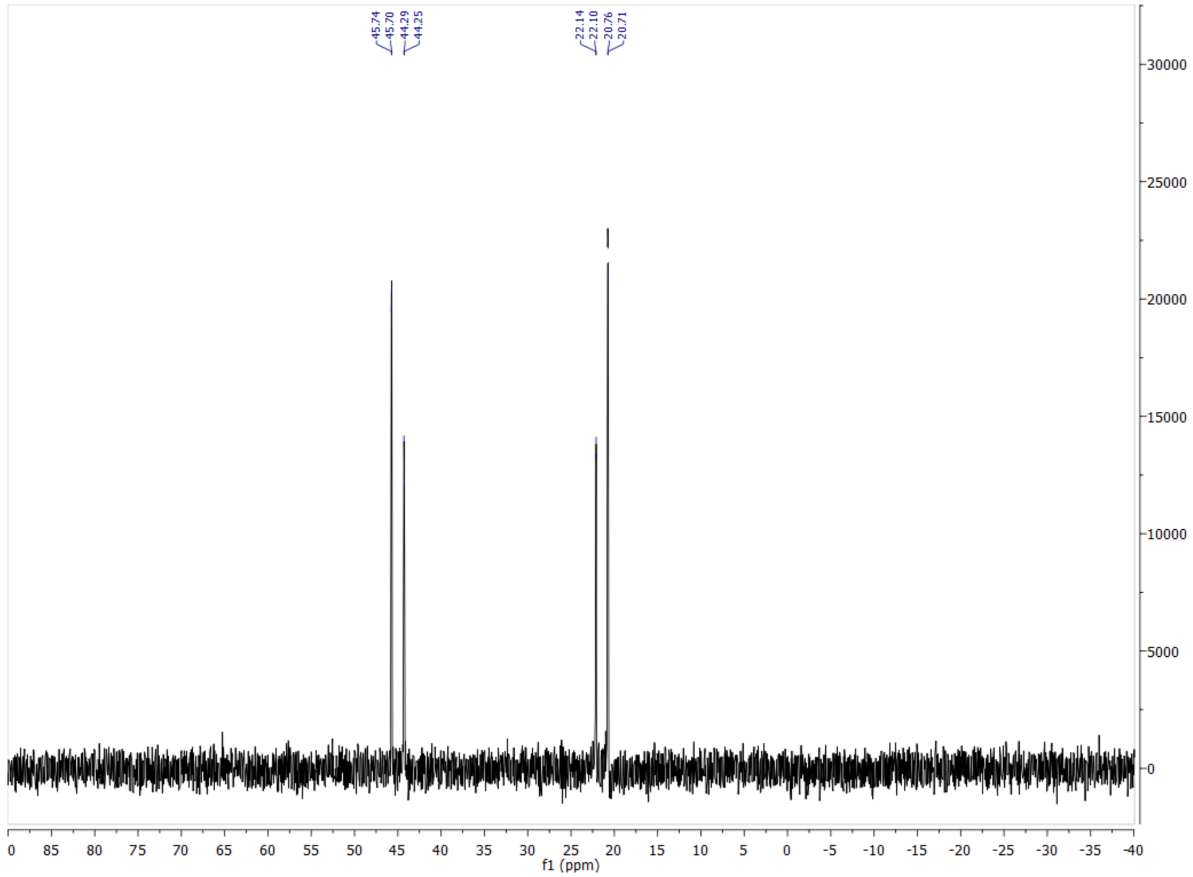

*Figure S7: $^{31}P\{^1H\}$-NMR spectrum of a mixture of the diastereoisomers **10** and **11** in CH_2Cl_2.*

^{195}Pt{^{1}H}-NMR Spectroscopy

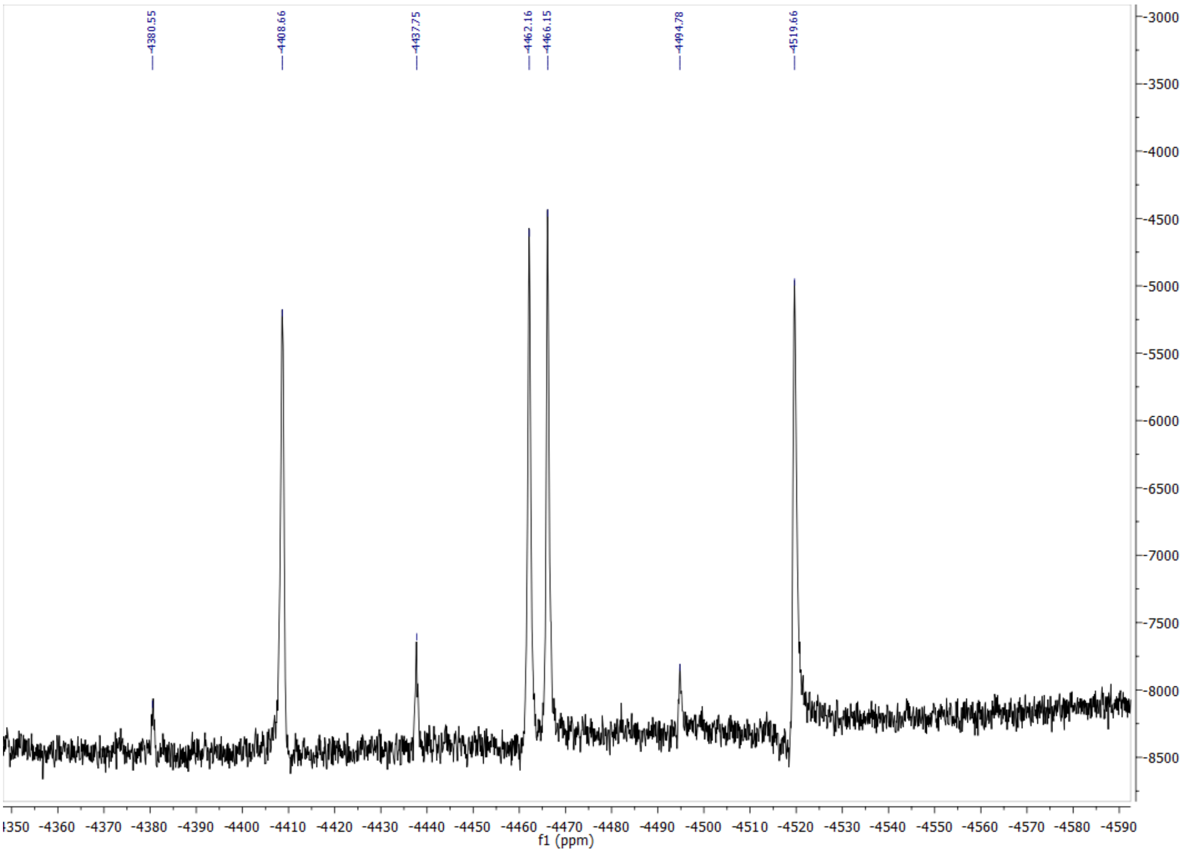

*Figure S8: ^{195}Pt{^{1}H}-NMR of **2** in DMF containing a small impurity of complex **3**.*
